# Supplementary material for: Antiangiogenic Compound Axitinib Demonstrates Low Toxicity and Antitumoral Effects against Medulloblastoma
Source: Cancers (Basel). 2021 Dec 24;14(1):70. doi: 10.3390/cancers14010070 (PMC8750527; doi:10.3390/cancers14010070)
Supplement: Supplementary file 1 [file cancers-14-00070-s001.zip › cancers-1477229-supplementary.pdf]

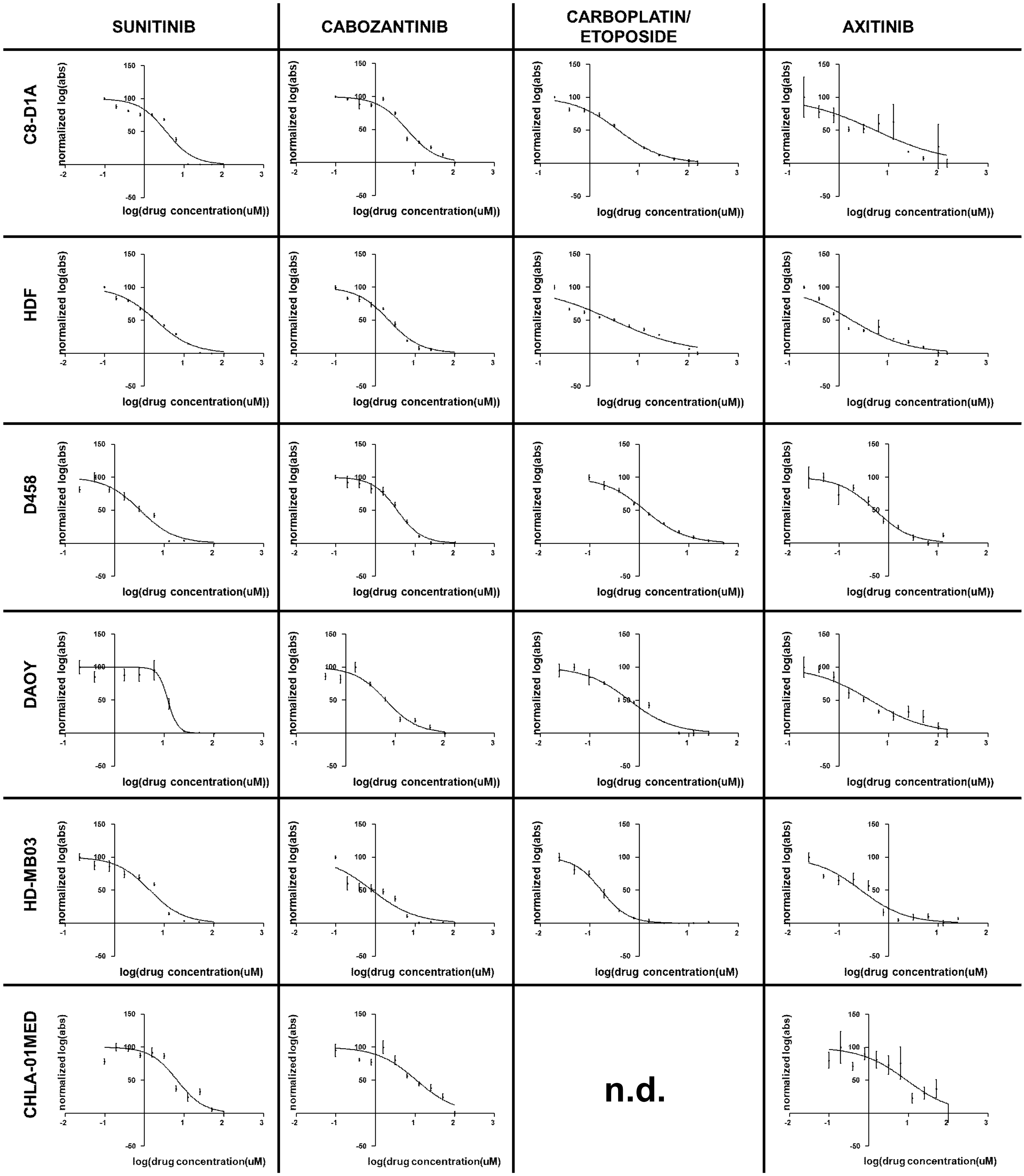

Supplementary Figure S1: Non-linear fit of MTT assay data. Graphs representing the normalized absorbance relative to drug concentration with various combinations of drugs (sunitinib, cabozantinib, carboplatin/etoposide, and axitinib) and cell types. Each data point on the graphs represents the mean +/-SEM of at least four technical replicates. A representative example of at least three experimental replicates is presented.

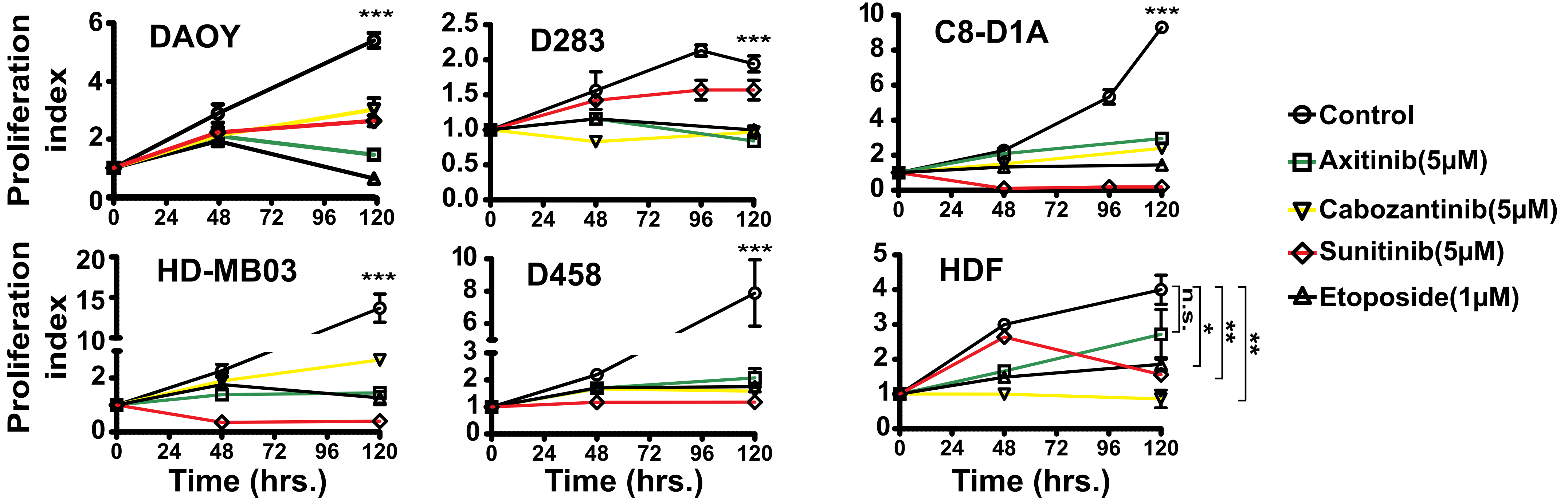

Supplemental Figure S2: Proliferation index of MB and normal cell lines continuously treated for 120 hours with the indicated concentrations of each compound (***: $p<0.001$, **: $p<0.01$, *: $p<0.05$, n.s.: non-significant, one-way Anova test comparing control conditions to the other experimental conditions).

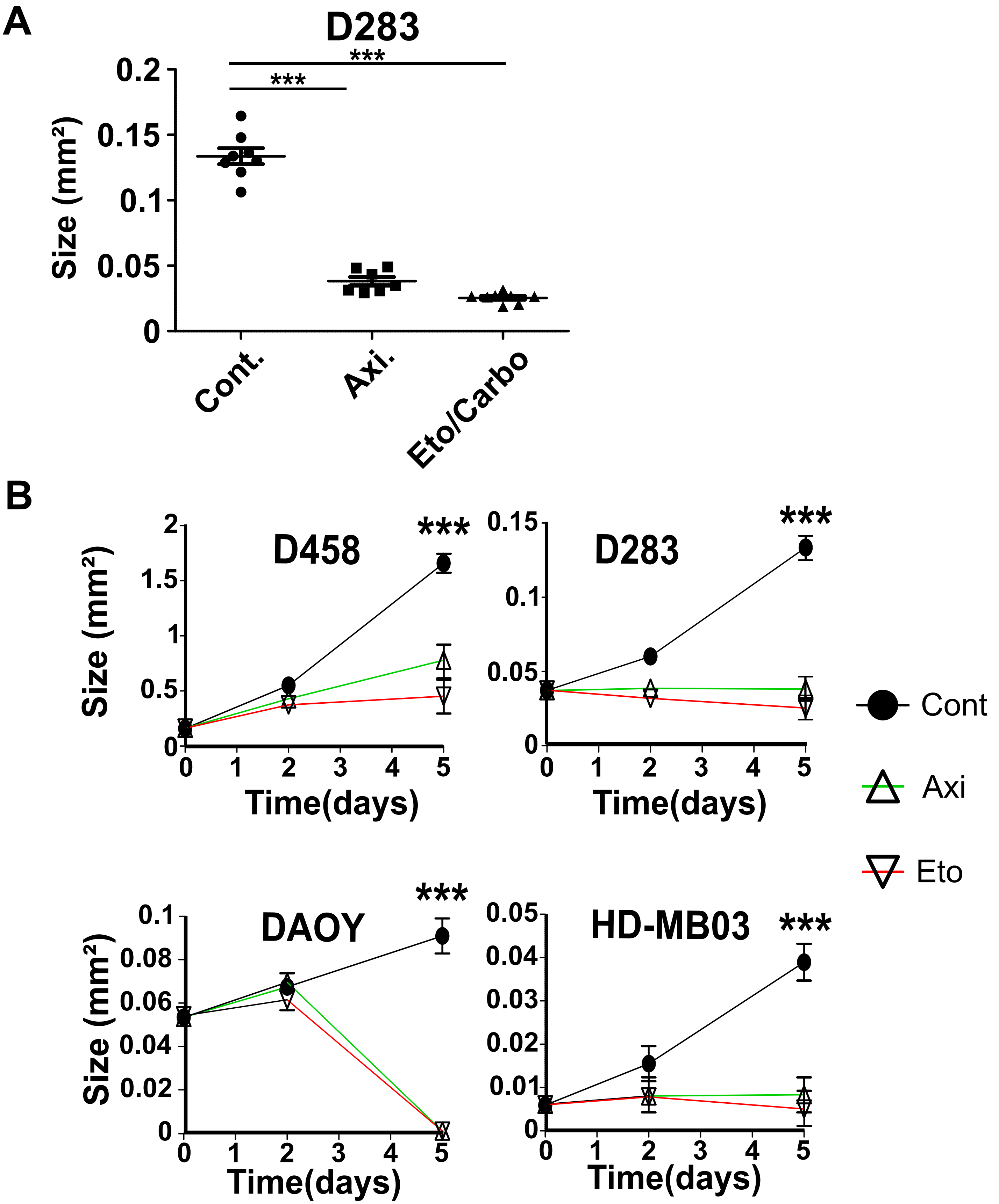

Supplemental Figure S3: Axitinib and etoposide reduce the growth of MB spheroids. (**A**) Dot plots showing the endpoint size measurements of spheroids generated with the D283 MB cell line and continuously treated with 5µM axitinib (Axi.) or 1µM etoposide (Eto). Controls were all treated with a concentration of DMSO corresponding to the one used as vehicle for the drugs. (**B**) Growth curves of spheroids generated with the indicated cell lines and treated as indicated above (results presented are mean +/- SEM from at least 3 independent experiments, ***: $p<0.001$, one-way ANOVA test).

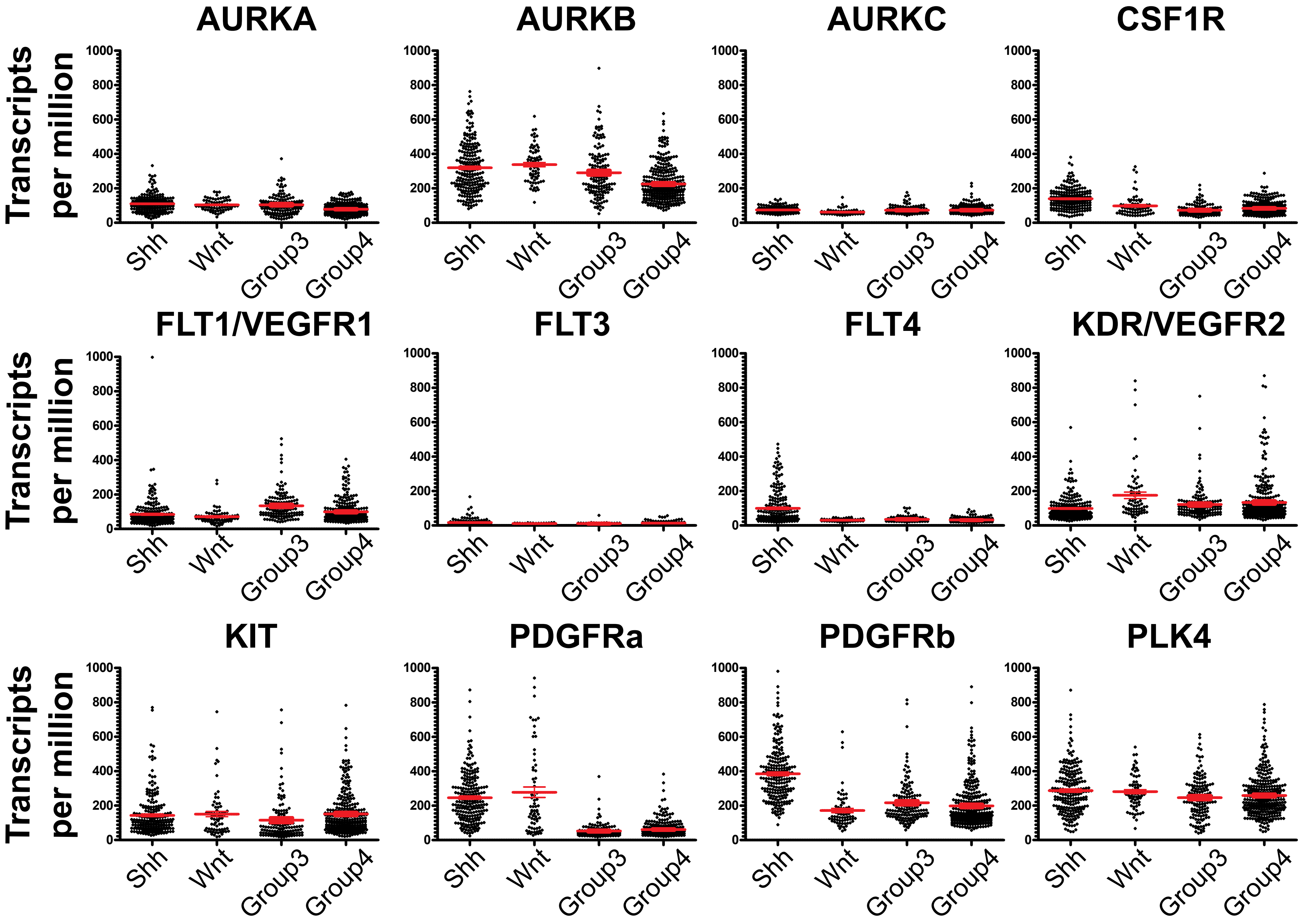

Supplementary Figure S4: Expression of axitinib kinase targets in MB cases. The expression level of axitinib described kinase targets across the four subgroups of MB was extracted from the transcriptomic data obtained by Cavalli and colleagues[27].

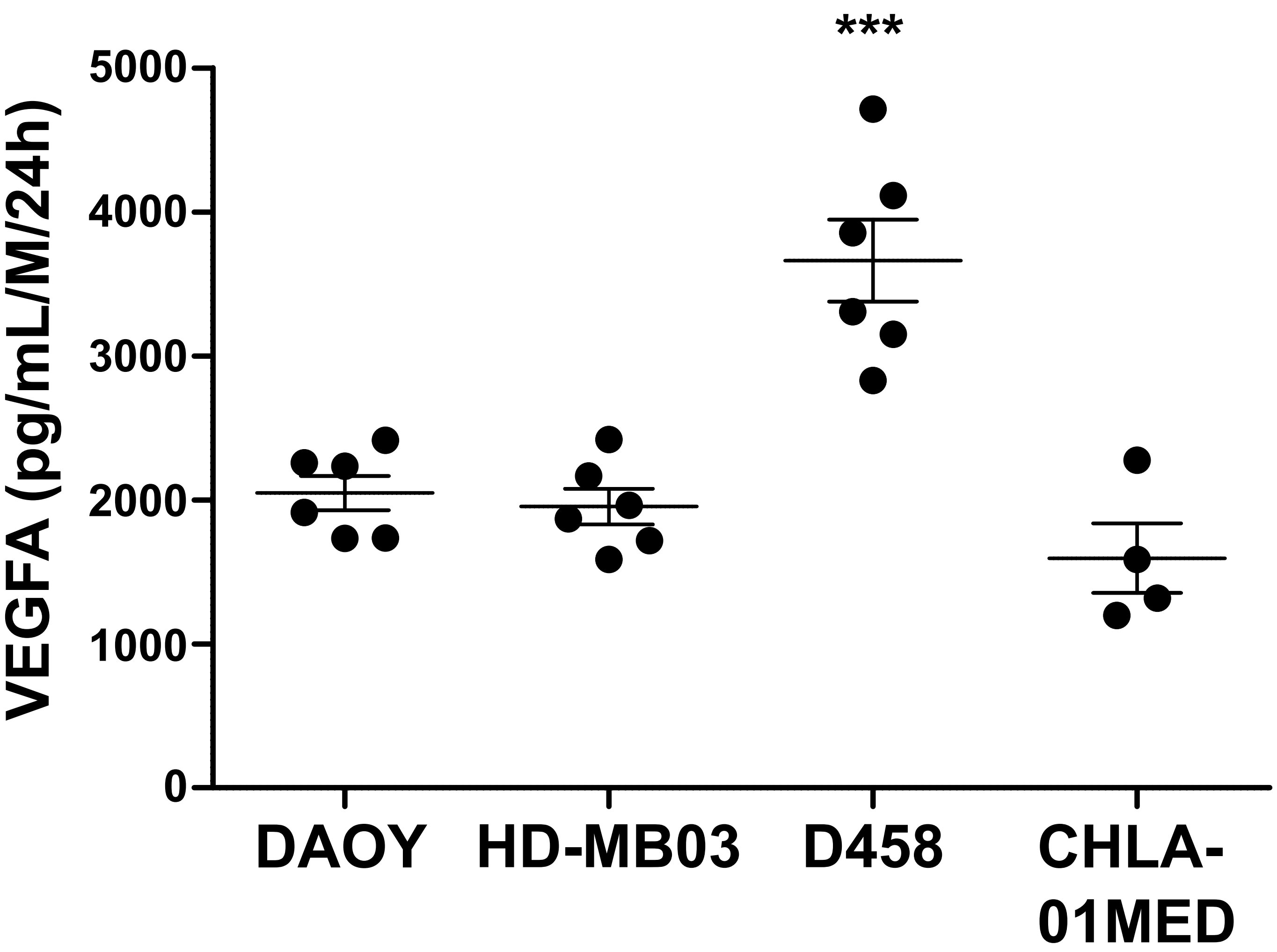

S**upplementary Figure S5: VEGFA expression in MB cell lines.** Dotplot presenting the expression level of VEGFA measured by ELISA in 2D cultures of four MB cell lines. The concentration level of VEGFA is expressed in pg per mL per million cells per 24 hours. (results presented are from at least 3 independent experiments, bars represent the mean +/- SEM, ***: $p<0.001$, one-way ANOVA test).

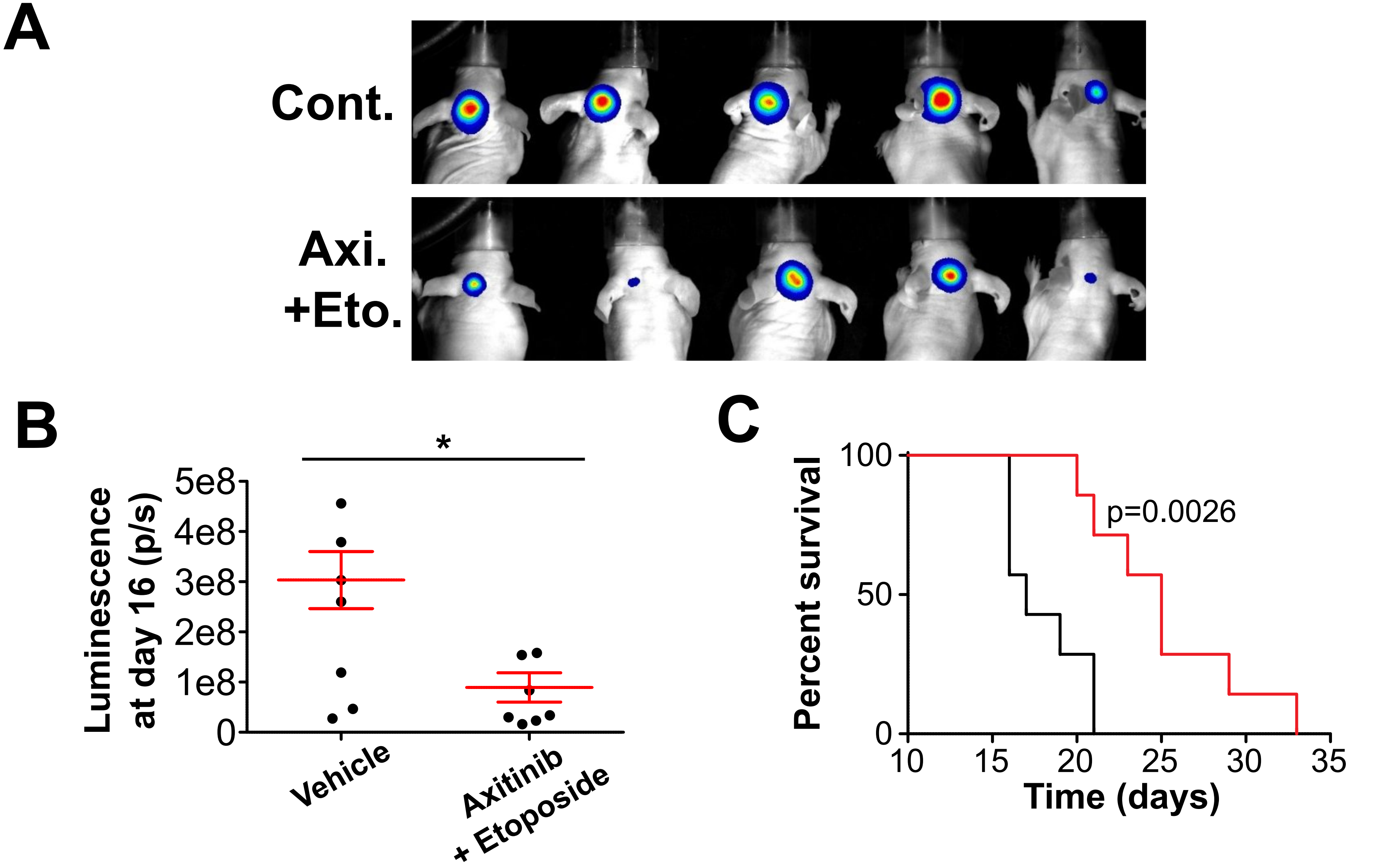

Supplementary Figure S6: Axitinib/etoposide combination reduce tumor growth and increase survival in an HD-MB03 orthotopic tumor model. (A) Luminescence image of mice after 16 days of orthotopical tumor growth (5 representative mice are presented for each treatment). (B) Quantification of the luminescence of the tumors presented in panel (B) (horizontal lines and errors bars represent the mean +/- SEM; *: p<0.05, Mann-Whitney test). (C) Survival curves of mice orthopically implanted with HD-MB03 in the cerebellum. Day 0 corresponds to the beginning of the treatment (black: control treatment; red: axitinib/etoposide combined treatment, p value of a Log-rank test is indicated).

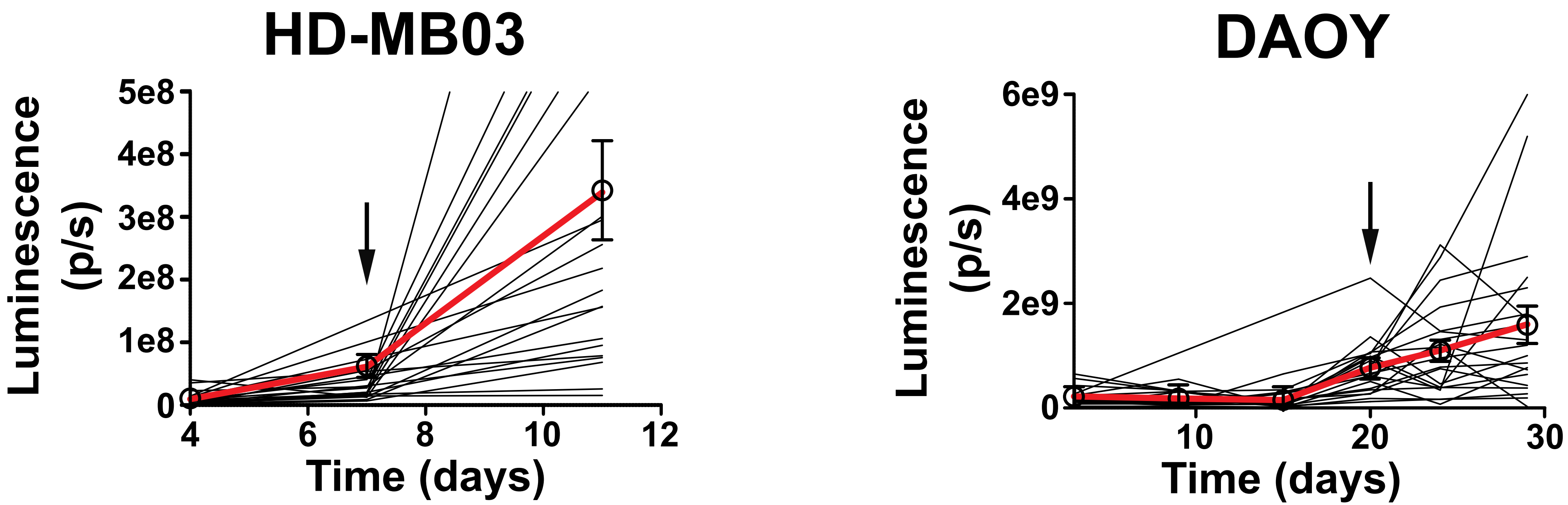

Supplementary Figure S7: Treatment onset decision based on luciferase activity in the tumors. Tumoral luminescence was measured twice weekly after injection of the cells. Randomization of the animals and beginning of the treatments (black arrows) were done when two consecutive measurements showed an increase (black lines are individual measurements for each animal; red lines are mean luminescence, error bars represent SEM).

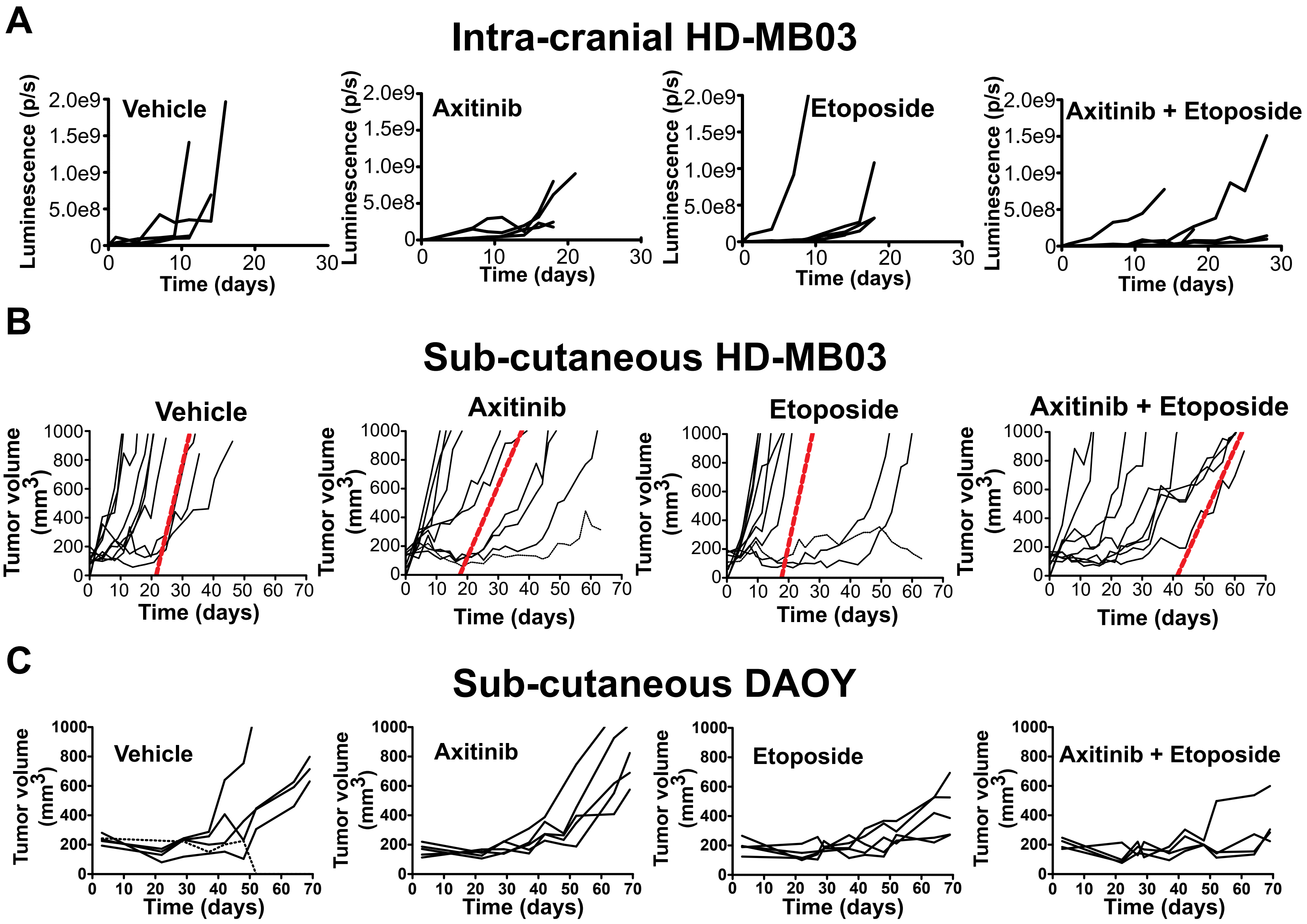

Supplemental Figure S8: Individual growth curves of experimental tumors. (**A**) The growth of intra-cranial tumors generated with HD-MB03 MB cells was measured by B.L.I. (**B**) The growth of sub-cutaneous tumors generated with HD-MB03 was measured based on the tumor volume. The mean growth rate estimated by linear regression method is represented on the graph by a dotted red line that crosses the x axis at the level of the median survival. Outliers are presented as a dotted black line. (**C**) The growth of sub-cutaneous tumors generated with DAOY was measured based on the tumor volume. Outliers are presented as a dotted black line.

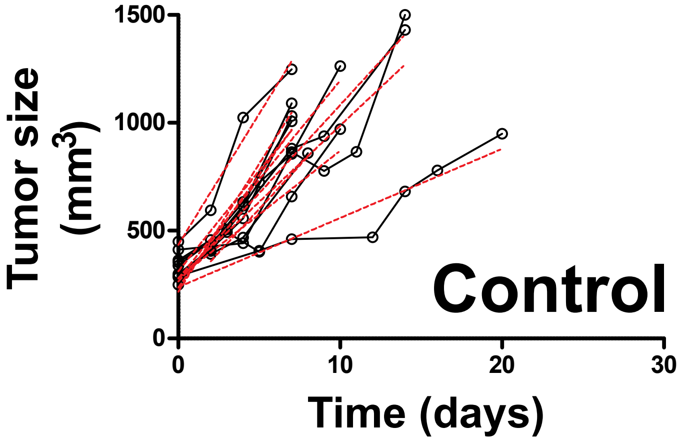

Control	
Slope	R square
104,5 ± 18,68	0.9399
91,86 ± 9,213	0.9707
121,3 ± 18,96	0.9534
80,77 ± 14,78	0.9676
93,42 ± 12,90	0.9633
32,05 ± 4,700	0.9029
118,6 ± 18,62	0.9759
81,76 ± 4,387	0.9914
70,74 ± 17,26	0.8077
58,94 ± 16,55	0.8087

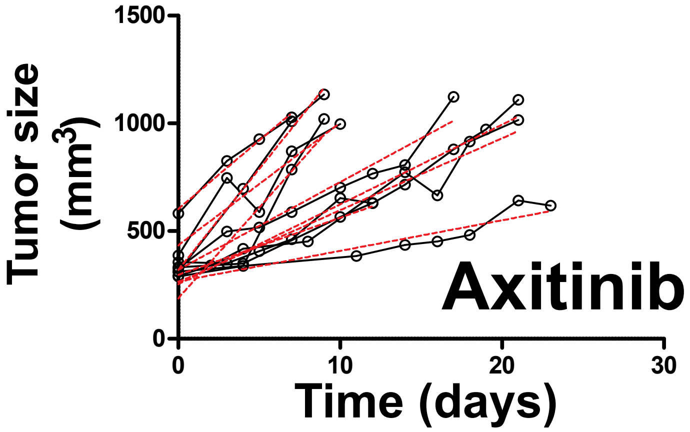

Axitinib	
Slope	R square
40,68 ± 4,233	0.939
37,02 ± 2,876	0.9539
26,52 ± 4,872	0.9368
33,40 ± 3,488	0.9386
63,55 ± 5,878	0.9832
14,19 ± 2,864	0.8307
84,17 ± 23,52	0.8649
56,86 ± 15,32	0.8212
93,75 ± 4,741	0.9949

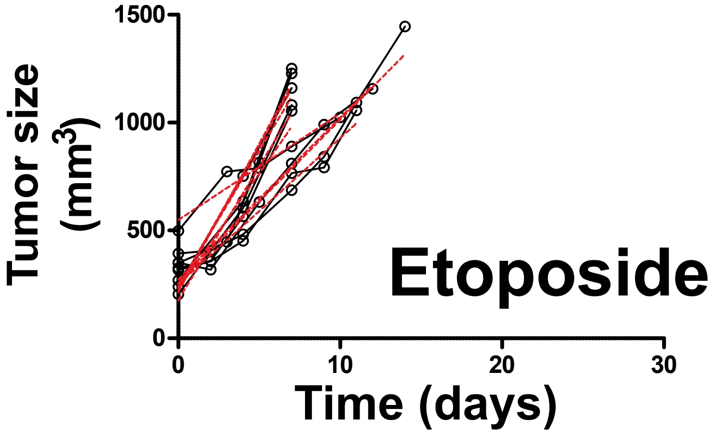

Etoposide	
Slope	R square
67,74 ± 7,525	0.953
75,76 ± 8,938	0.9349
107,4 ± 23,83	0.9103
75,08 ± 7,027	0.9661
137,6 ± 31,94	0.9489
125,8 ± 32,15	0.8844
131,4 ± 2,392	0.9997
49,41 ± 7,098	0.9417
123,9 ± 16,57	0.9824

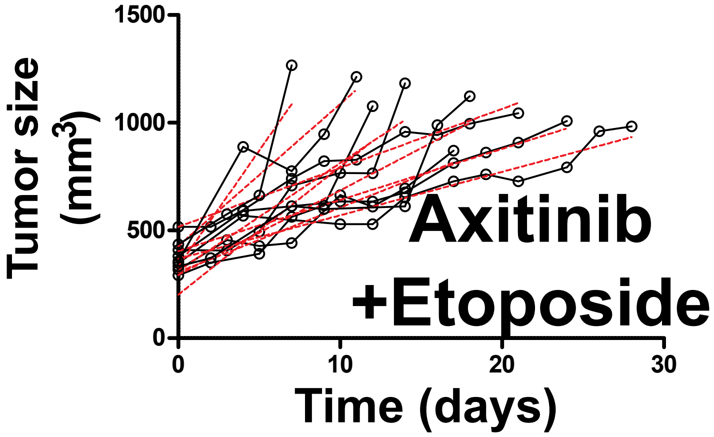

Axitinib + Etoposide	
Slopes	R^2
29,89 ± 3,802	0.9115
50,01 ± 9,740	0.8406
27,37 ± 2,006	0.9588
20,16 ± 2,293	0.8957
23,71 ± 2,193	0.9435
59,41 ± 12,73	0.8448
65,92 ± 17,13	0.8316
39,20 ± 7,417	0.8482
107,8 ± 41,62	0.7702

Supplementary Figure S9: Tumor growth rate evaluation by a linear regression method. Growth curves were generated for each individual HD-MB03 subcutaneous tumor starting from the first measurement over 200mm^3. A linear regression fit method was used to estimate the average growth rate of each tumor (estimated slopes+/- SEM). The corresponding R2 values are presented in the table aside the corresponding growth curves).

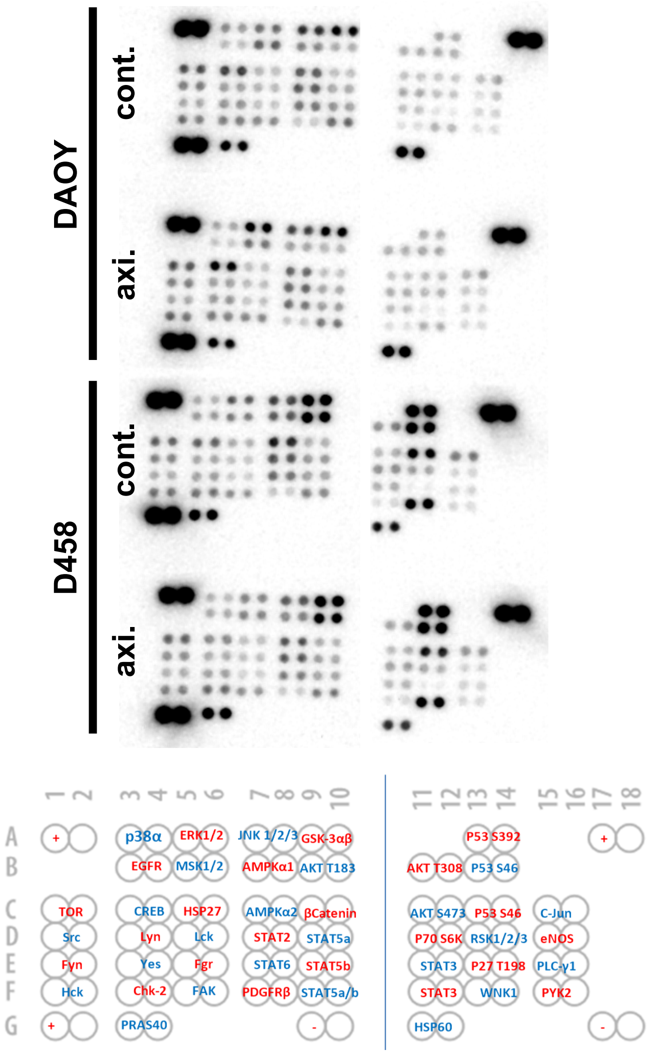

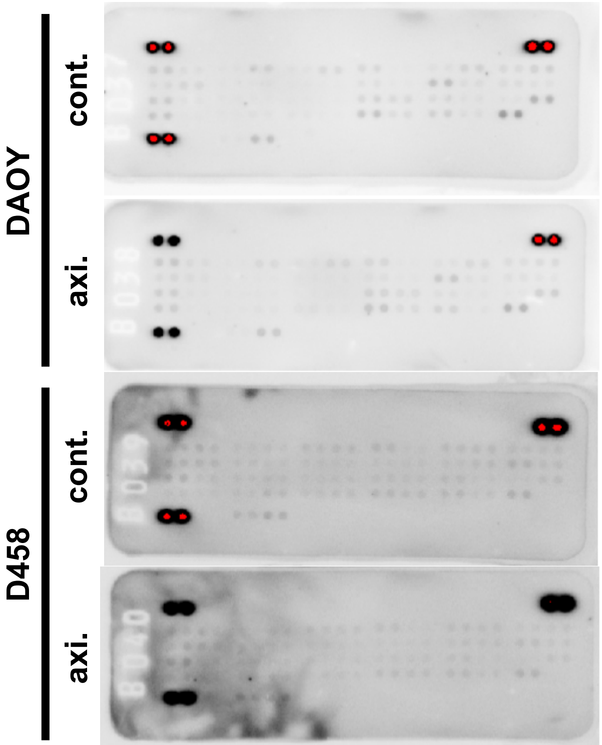

Coordinate	Receptor Family	RTK/Control	Coodinate	Receptor Family	RTK/Control
A1, A2	Reference Spots	——	D1, D2	Tie	Tie-2
A23, A24	Reference Spots	——	D3, D4	NGF R	TrkA
B1, B2	EGF R	EGF R	D5, D6	NGF R	TrkB
B3, B4	EGF R	ErbB2	D7, D8	NGF R	TrkC
B5, B6	EGF R	ErbB3	D9, D10	VEGF R	VEGF R1
B7, B8	EGF R	ErbB4	D11, D12	VEGF R	VEGF R2
B9, B10	FGF R	FGF R1	D13, D14	VEGF R	VEGF R3
B11, B12	FGF R	FGF R2α	D15, D16	MuSK	MuSK
B13, B14	FGF R	FGF R3	D17, D18	Eph R	EphA1
B15, B16	FGF R	FGF R4	D19, D20	Eph R	EphA2
B17, B18	Insulin R	Insulin R	D21, D22	Eph R	EphA3
B19, B20	Insulin R	IGF-I R	D23, D24	Eph R	EphA4
B21, B22	Axl	Axl	E1, E2	Eph R	EphA6
B23, B24	Axl	Dtk	E3, E4	Eph R	EphA7
C1, C2	Axl	Mer	E5, E6	Eph R	EphB1
C3, C4	HGF R	HGF R	E7, E8	Eph R	EphB2
C5, C6	HGF R	MSP R	E9, E10	Eph R	EphB4
C7, C8	PDGF R	PDGF Rα	E11, E12	Eph R	EphB6
C9, C10	PDGF R	PDGF Rβ	E13, E14	Insulin R	ALK
C11, C12	PDGF R	SCF R	E15, E16	——	DDR1
C13, C14	PDGF R	Flt-3	E17, E18	——	DDR2
C15, C16	PDGF R	M-CSF R	E19, E20	Eph R	EphA5
C17, C18	RET	c-Ret	E21, E22	Eph R	EphA10
C19, C20	ROR	ROR1	F1, F2	Reference Spots	——
C21, C22	ROR	ROR2	F5, F6	Eph R	EphB3
C23, C24	Tie	Tie-1	F7, F8	——	RYK
			F23, F24	Control (-)	PBS

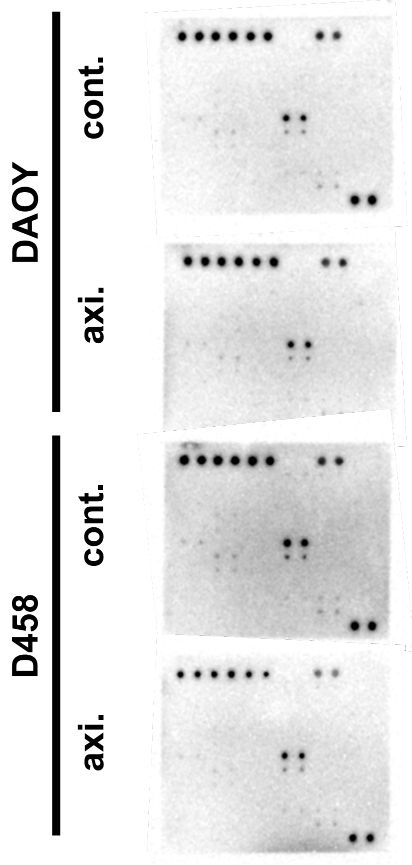

XI. ARRAY MAP

	A	B	C	D	E	F	G	H	I	J	K	L
**1**	POS1	POS1	POS2	POS2	POS3	POS3	ABL1	ABL1	ACK1	ACK1	ALK	ALK
**2**	NEG	NEG	NEG	NEG	Axl	Axl	Blk	Blk	BMX	BMX	Btk	Btk
**3**	Csk	Csk	Dtk	Dtk	EGFR	EGFR	EphA1	EphA1	EphA2	EphA2	EphA3	EphA3
**4**	EphA4	EphA4	EphA5	EphA5	EphA6	EphA6	EphA7	EphA7	EphA8	EphA8	EphB1	EphB1
**5**	EphB2	EphB2	EphB3	EphB3	EphB4	EphB4	EphB6	EphB6	ErbB2	ErbB2	ErbB3	ErbB3
**6**	ErbB4	ErbB4	FAK	FAK	FER	FER	FGFR1	FGFR1	FGFR2	FGFR2	FGFR2 (α isoform)	FGFR2 (α isoform)
**7**	Fgr	Fgr	FRK	FRK	Fyn	Fyn	Hck	Hck	HGFR	HGFR	IGF-IR	IGF-IR
**8**	Insulin R	Insulin R	Itk	Itk	JAK1	JAK1	JAK2	JAK2	JAK3	JAK3	LCK	LCK
**9**	LTK	LTK	Lyn	Lyn	MATK	MATK	M-CSFR	M-CSFR	MUSK	MUSK	NGFR	NGFR
**10**	PDGFR-α	PDGFR-α	PDGFR-ß	PDGFR-ß	PYK2	PYK2	RET	RET	ROR1	ROR1	ROR2	ROR2
**11**	ROS	ROS	RYK	RYK	SCFR	SCFR	SRMS	SRMS	SYK	SYK	Tec	Tec
**12**	Tie-1	Tie-1	Tie-2	Tie-2	TNK1	TNK1	TRKB	TRKB	TXK	TXK	NEG	NEG
**13**	Tyk2	Tyk2	TYRO10	TYRO10	VEGFR2	VEGFR2	VEGFR3	VEGFR3	ZAP70	ZAP70	POS4	POS4

Supplementary Figure S10: Antibody arrays for the screening of potential axitinib targets in MB cells. Several antibody arrays spotted with antibodies targeting phosphorylated proteins were used to screen for phosphorylation inhibition in DAOY and D458 cells treated with 10µM axitinib over 24 hours.

Supplementary Method

Tumor Volume

The data used in this document is stored in the files

- 'data/DAOY.csv'
- 'data/HDMB.csv'

DAOY

Data Manipulation The experiment compares 3 treatments (**T1**, **T2**, **T3**) versus a control group (**C**). Each of the cases consist on n individuals (between 4 and 5 depending on the case) that have tumoral cells. At the same moment of injection of tumoral cells, the treatment is also applied (except for control). The total tumoral volume is measured using images and an ellipsoid approximation for all individuals at several moments (all measurements are simuktaneous). If the tumoral mass pass a given limit, then the individual is sacrified, which creates non random missing data. (*enders reference*). As imputation method, we simply replace the growth of rate of the last period for the missing data by the mean growth rate for all the individuals in the same group at the same period. Notice that we are implicitly assuming that the tumor growth rates are independent of the tumoral mass which is probably too simplistic, but within the range of observation it seems reasonable.

This imputation is only needed in one occasion, for an indiviudual in the control group that was sacrified before the experiment ended, and the last volume measurement is missing.

The following graph shows the curves for the volume of tumors. (Black=control. Red, Green, Blue=Treatments 1, 2, and 3 respectively). The starting point have been normalized as $V(t = 0) = 1$. The thick lines correspond to the (average values of the normalized curves of each group.

```
source('tumorVolume.R')
generate_curves(DAOY)
```

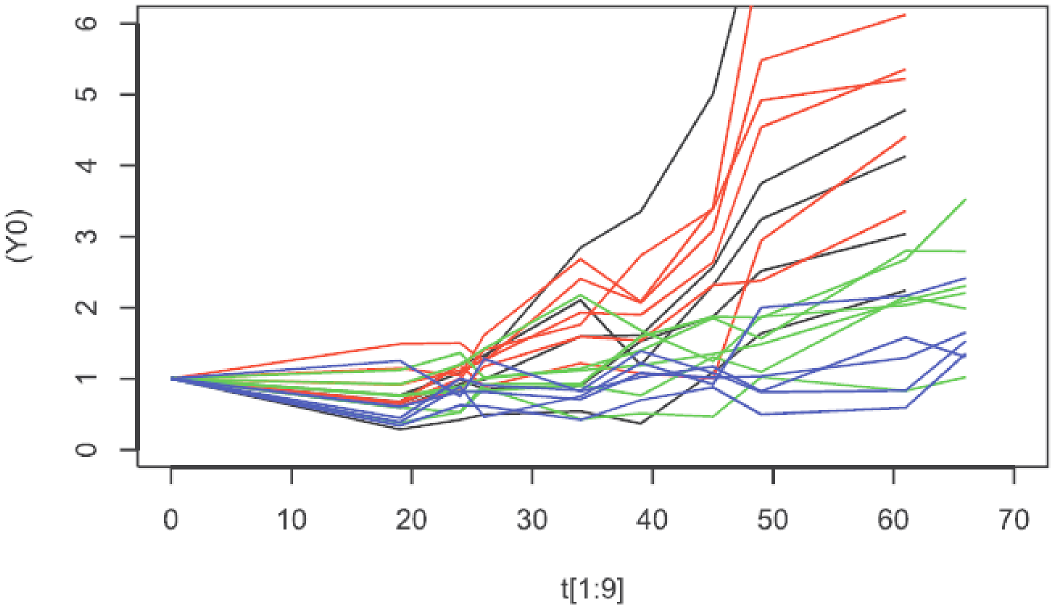

At first sight, it seems that for all treatmments, the initial response is decreasing and it is only after the 19th day measurement that the volume increase exponentially (at different rates depending on the treatment. We

assume that after the first 19-days period, each of the processes follow a lognornal model as:

$$V^{T+\Delta t} - V^T = \mu_\alpha V^T \Delta t + \sigma_\alpha V^T \Delta W$$

where ΔW are independent increments of a brownian process (Gaussian noise) with variance ΔT, and $(\mu_\alpha, \sigma\alpha)$ which encode the rates of growth and the variances of the volume measurements, depend on the treatments

$$\alpha \in (\text{Control, Treat1, Treat2 Treat3}).$$

The solution for such lognormal process is given by the equation

$$V(T) = V(0)e^{\mu^*_\alpha T + \sigma_\alpha \int dW}$$

where

$$\mu^*_\alpha = \mu_\alpha - \frac{1}{2}\sigma^2_\alpha$$

This means, that the logarithm of the ratio between the volume and th initial condition, should increase limerly with time.

$$ln\left(\frac{V_{i+1}}{V_i}\right) = \mu^* \Delta T + \sigma \Delta W$$

where $V_{i+1} = V(T_{i+1})$, $V_i = V(T_i)$, and $\Delta T = T_{i+1} - T_i$.

To test visually if this is reasonable, we plot also the logarithm of the volumes (same color code as first graph) to see if the slopes are linear.

```
generate_logcurves(DAOY)
abline(v=19, col='grey70')
```

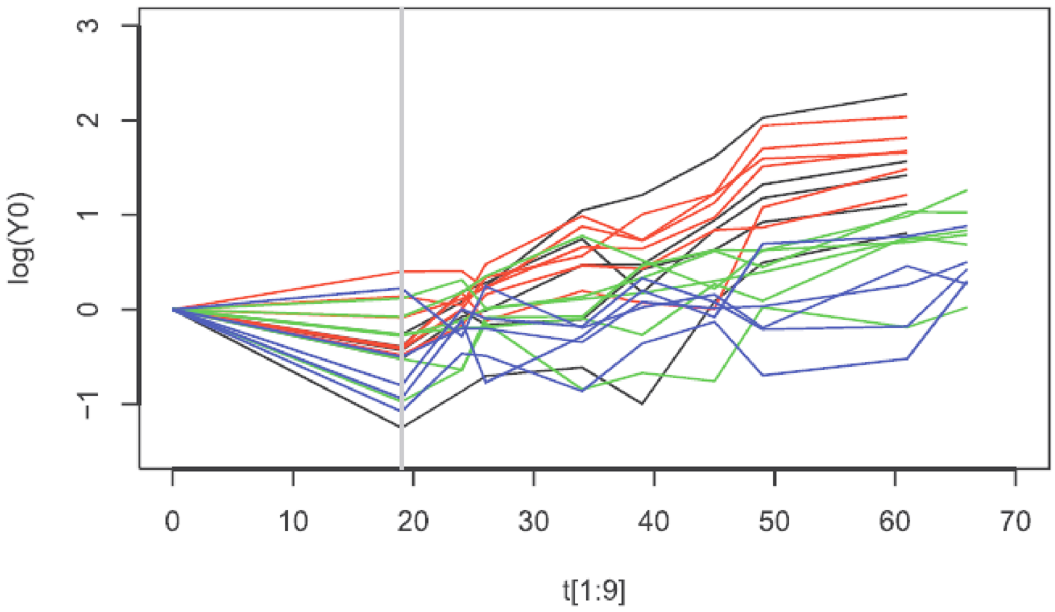

Control and treatment 1 (black and red respectively) appear to have very similar growth rates, while treatments 2 and 3 (blue and green respectively) share a smaller growth rate, Treatment 3 appears to be less stable (higher variance) that treatment 2, althogh treatment 3 decreased more iun the first periond of treatment, and the differences at the end of the treatment are all explained by the differences in the initial 19 days.

GLM

We perform then a Generalized Linear Model analysis for each of the treatments, with the individuals as fixed factors

$$ln\left(\frac{V_T^j}{V_0^j}\right) = \mu T + \sigma \Delta W^j + \alpha^j$$

where $j = 1, .., 4$ are the individuals. We also perform the same analysis with the hypothesis that the intercept is the same for all individuals. The results of these two models are given in the following table.

GLM with individuals as fixed factors

```r
kable(result_glm_fixed)
```

treatment	mean	sd	0.50%	2.50%	median	97.50%	99.50%	ind1	ind2	ind3	ind4	ind5
C	0.045	0.004	0.034	0.036	0.045	0.054	0.057	-	**			
T1	0.043	0.004	0.034	0.036	0.043	0.050	0.052	-	*	.	**	***
T2	0.022	0.003	0.014	0.016	0.022	0.027	0.029	-		*		
T3	0.015	0.004	0.005	0.007	0.015	0.023	0.025	-		***	*	

GLM without fixed factors.

```r
kable(result_glm)
```

treatment	mean	sd	0.50%	2.50%	median	97.50%	99.50%
C	0.045	0.006	0.030	0.034	0.045	0.056	0.060
T1	0.043	0.005	0.031	0.034	0.043	0.052	0.055
T2	0.022	0.004	0.013	0.015	0.022	0.029	0.031
T3	0.015	0.007	-0.003	0.001	0.015	0.029	0.033

These results suggest that T2 and T3 reduce significantly the growth of tumor volume, beong the effects of T3 significanly better than T2.

The estimates for the rate of growth is practically equivalent when considering the individuals as fixed factors or not, but the confidence intervals are wider in the second model, as it is expected.

The interpretation of a fixed factor per individual is that althought we are taking a starting time of 19 days as the moment in which the tumors growth at their normal rate, (compared to the initial latent period in which the growth of rate is much smaller), there is some uncertainty about the exact moment in which the tumor volume starts growing exponentially. If for some individual, the latent period extends beyond our reasonable but nevertheless arbitrary threshold of 19 days, then we would need to add a fixed factor to take into account for a non null intercept.

If this interpretation is correct, one should exect a disminishing of relevance of the fixed factors as the threshold is taken at a further time. *(shoud I test this?)*
